# Is Head and Neck Resection of the Femur (Girdlestone’s Procedure) Still Relevant? Indications and Results About 24 Cases

**DOI:** 10.2174/1874325001812010069

**Published:** 2018-02-28

**Authors:** Mamoudou Sawadogo, Hamado Kafando, Salam Ouedraogo, Alexandre Stanislas Korsaga, Souleymane Ouedraogo, Sayouba Tinto, Anatole Jean Innocent Ouedraogo, Mohamed Tall, Songahir Christophe DA

**Affiliations:** 1 Yalgado Ouedraogo University Hospital 03 BP 7022 Ouagadougou 03 Burkina Faso; 2 Ouahigouya Regional University Hospital Center BP 36, Ouahigouya Burkina Faso

**Keywords:** Resection, Head, Neck, Femur, Procedure, Girdlestone

## Abstract

**Introduction:**

Head and neck resection of the femur was described by Girdlestone in 1928 in the treatment of coxalgia. Very invasive at the beginning, this intervention is much less so today, but the term of “Girdlestone’s operation” or “Girdlestone’s procedure” has remained in use. The reported results are controversial. In resource-limited countries, Girdlestone’s procedure is often indicated for lack of a better one. In this context, we report the results of a series of 24 patients operated in a regional hospital (Ouahigouya, Burkina Faso) with the aim of showing that this technique remains valid and can be benefit.

**Methods:**

This was a retrospective descriptive study of 24 patients who had benefited from the procedure for cervical fracture sequelae, failure of arthroplasty or osteosynthesis, or osteonecrosis. All were operated by posterolateral approach, under spinal anesthesia and followed for 5 years with evaluation of the anatomical and functional results using the rating of Postel and Merle d'Aubigné (PMA).

**Results:**

All patients had Trendelenburg lameness with a mean shortening of 3.5 cm. They were all autonomous with walking aids and the PMA score ranged from 16 to 14. Discussion: although the results obtained are not excellent, they are relatively good and have allowed all our patients to recover an acceptable autonomy, compatible with certain independence in everyday life.

**Conclusion:**

The Girdlestone’s procedure cannot be a first intention indication, but retains a place in the therapeutic arsenal of certain affections of the hip.

## 1 INTRODUCTION

The first head and neck resection of femur was performed in 1818 by Anthony White in a 9-year-old child with septic pseudarthrosis of the hip. However, the procedure was popularized by Gathorne Robert Girdlestone [1] who reported his technique in 1928 in the treatment of tuberculosis of the hip. His technique was very invasive at first and consisted of a transverse approach of the hip with resection of a large portion of the gluteal muscles as well as the greater trochanter, resection of the neck and femoral head and removal of all acetabular cartilage. Sometimes partial

resection of adductors and pectin, and release of neurovascular structures were associated. The wound was then left in directed healing. Current interventions are much less invasive, but the term of “Girdlestone procedure” has remained. The results of this intervention reported in the literature remain controversial. This study evaluates the functional outcome of 24 patients undergoing a gridlestone procedure after a minimum follow up of 5 years.

## 2 MATERIALS AND METHODS

Twenty-four patients were operated at Ouahigouya Regional Hospital between 2011 and 2015. There were 11 women and 13 men. The average age at the time of the procedure was 67.5 years (Range 28 to 113 years). The average BMI was 20.1 (range 19.7 to 26.4). Fourteen patients underwent the girdlestone procedure for negleted femoral neck fracture (traditionnal treatment), 3 for failed osteosynthesis, 2 for failed hemi-arthroplasty (Moore's prosthesis without cement in all cases), and 5 for painful aseptic osteonecrosis of the femoral head in patients who could not afford arthroplasty_._ All patients were operated by posterolateral approach under spinal anesthesia. The study included 11 right and 13 left hips. In cases of painful pseudarthrosis of the neck and complications of osteosynthesis (suppuration and / or early disassembly of material) or arthroplasty the level of section on the femur corresponded to the fracture line. We were simply performing a regularization of the fracture surface of the neck. In case of sepsis, debridement-lavage was associated. In aseptic osteonecrosis of the femoral head (AON), the neck was cut 1.5 cm from the small trochanter.

There was no complementary gesture on the acetabulum or tenotomy. We did not perform postoperative traction. No specific postoperative rehabilitation was provided due to the lack of appropriate structures: no physiotherapy service in the region, no follow-up care and rehabilitation. Patients on leaving the hospital returned to their homes. They were encouraged to crutch and mobilize the hip. They were periodically reviewed and the therapeutic results were evaluated based on anatomical criteria (shortening and radiographic aspects), and functional according to the rating of Postel and Merle d'Aubigné (PMA) [2].

## 3 RESULT

At the average follow-up of 5 years, we noted:- Mortality: 5 patients died or 20.8% of the workforce. Deaths occurred between one and 42 months (mean = 13.3 months) for undetermined reasons.- Anatomical aspects: the shortening of the limb varied from three to six centimeters with an average of 3.5 cm Fig. (**1**). There was an average amyotrophy of the thigh of 1.5 cm (extremes of 0.5 and 3 cm). On control radiographs there was no contact between the proximal femur and the pelvis. No significant periarticular ossification was noted Fig. (**2**). Five patients had died and five had been lost to follow-up. The functional results were therefore evaluated in 14 patients, five women and nine men of middle age = 57.1 years (range 28 to 83 years).- The pain: it was weak in three patients, moderate and episodic among the 11 others. No patient was taking medication regularly.- Autonomy: all patients were autonomous with the following PMA scores (Table **1**). Depending on the indication, the score obtained is shown in Table (**2**). Score 16 involved a 31-year-old female patient with an aseptic osteonecrosis of the left femoral head. Three other patients aged 28, 37 and 38 had a score of 15. They could do without any help walking on short distances and at home. Ten out of 14 patients (71.4%) used a single crutch and four of them used two (28.6%).- The mobility of the hip Fig. (**3** and **4**): the average flexion was 102° and was distributed as follows: 90 to 100°: seven patients100 to 110°: three patients110 to 120: four patients.

## 4 DISCUSSION

The current paper shows reasonable functional results in patients undergoing Girdlestone’s procedures. While patients had an average leg length discrepancy of 3.5 cm and displayed a positive Trendelenburg sign all patients were able to ambulate with walking aids. Epidemiological aspects: the average age of the patients at the time of surgery was 67.5 years (range 28 to 113 years). These ages correspond to those found by various authors such as Haw [3], Clegg [4], Bourne [5], or Lowry [6]. Three types of indication were selected: painful nonunion of the femoral neck, osteonecrosis of the femoral head and complications of arthroplasty or osteosynthesis. These were indications of necessity in a context of socio-economic precariousness characterized by a deficiency of the technical platform, a low income of the patients and a lack of health cover. Mortality at the 5-year follow-up was 20.8%, comparable to other authors such as Bourne [5], Sharma H [7], Schröder [8], or Lowry [9] and relationship with the relatively high average age of patients in different series.The average shortening of the limb was 3.5 cm. Various authors report values ​​between two and 13 cm [3-6, 9]. This shortening is inevitable and important. It is not influenced by postoperative traction [5, 6]. The patient must be notified before the procedure. However according to Lowry [6] there is no relationship between the importance of shortening and functional abilities. Complementary gestures have been proposed by some authors in order to preserve the mobility of the hip. Thus, proximal femur angulation osteotomy was performed in two stages by Batchelor [10] in 1948, and then in a single step by Milch [11] in 1959. Resection of the acetabular rims was proposed by Scott [12] and the interposition of flaps of soft tissue by Nelson [13]. We have not realized any of these artifices because they have not proved their utility as Haw [3], Taylor [14], Schepherd [15], Parr [16] or Murray [17] has shown.

Functionally, the 14 patients assessed were all autonomous with a PMA score ranging from 14 to 16, and showing fairly good results. These results are similar to those of Bourne [5], Lowry [6] and Schröder [8]. Conversely, other authors report poor functional results: Clegg [4], Hudec [18], and Ralf [19]. Regarding the use of walking aids, ten patients (71.4%) with a mean age of 51 years used a single crutch and four (28.6%) with a mean age of 68 years used two. Young age therefore appears as a factor of good prognosis. This is consistent with the findings of Haw [3] and Lowry [6]. But young subjects today satisfied, could they eventually deteriorate later? On the contrary, we observed an improvement in the PMA score in three elderly patients (70, 82, 83 years), from 12 postoperatively to 15 after two years. Shepherd [15] made the same observation of improvement of the functional result over time.The average flexion of the hip was 102° and allowed all the usual movements. This average, much higher than what is reported [3, 4, 6], could be explained by the living conditions in our villages where there is no special arrangement for people with reduced mobility. Patients are forced to sometimes extreme gestures such as squatting for toilets, and at the same time reeducate their articulation which ends up acquiring a significant mobility. Regarding the subjective perception of the result, all our patients said they were satisfied. But this rate must be nuanced in the traditional African context and religious where the acceptance of its situation is of rule: “It is the will of God”. However, even in the literature, this rate is quite high: 60 to 91% [3, 5, 6, 20], even in authors who report poor functional results [18]. Ultimately, even if the functional abilities of patients are not excellent, no author reports results frankly bad.

## CONCLUSION

With the progress of modern orthopedics, the Girdlestone’s resection is rarely indicated as a primary procedure at the outset. However, it retains a place in the arsenal of surgical hip techniques. It is a simple procedure giving a mobile, painless and stable hip, at the cost of a shortening. This is sometimes the only indication for septic complications. In our unfavorable socio-economic context characterized by a deficit of technical plateaus in particular in the field of arthroplasty, the indications of Girdlestone are broader. The results reported in the literature without being excellent, are rarely really bad. They allow in most cases a certain degree of functional autonomy compatible with an acceptable daily life. In our series, the final results are more than satisfactory and have allowed young people in particular to return to an active life.

## Figures and Tables

**Fig. (1):**
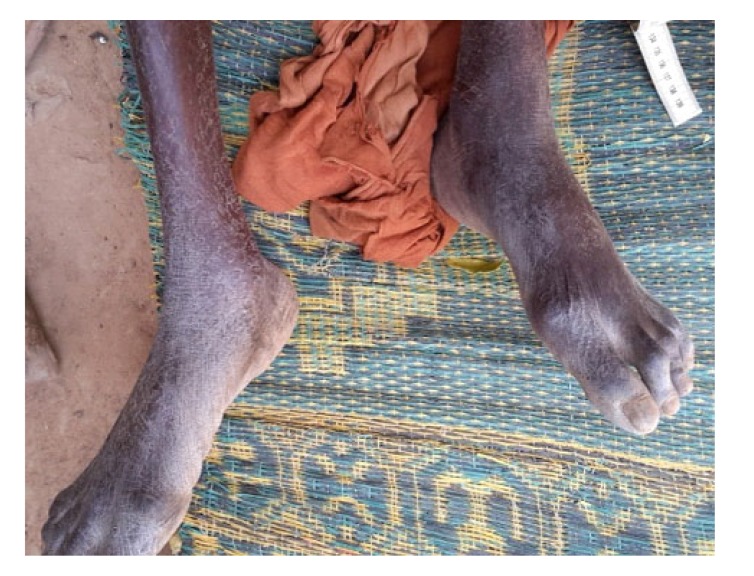
Shortening view in a 83 old-years patient.

**Fig. (2):**
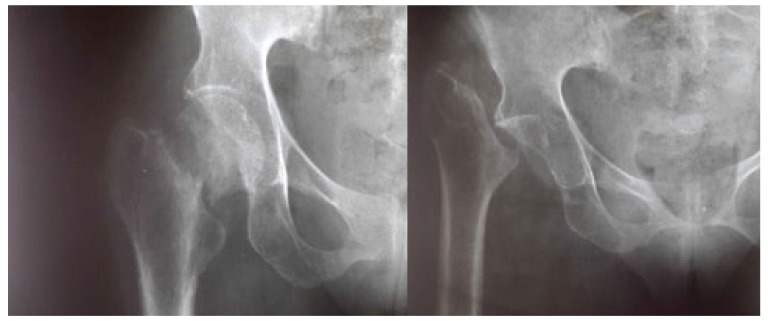
Control X-rays after two years: no contact between the proximal femur and the pelvis and no periarticular ossification was noted.Table (**1**): Breakdown by lesion diagnosis.

**Fig. (3):**
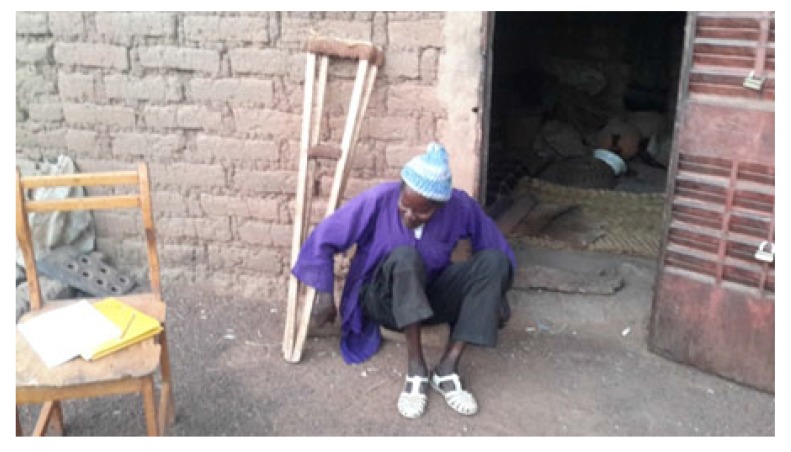
Patient of 82 years with flexion allowing to sit on a low seat.

**Fig (4):**
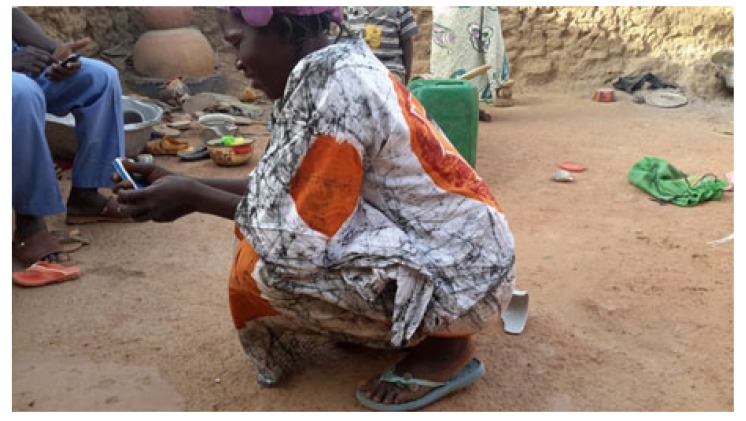
Patient of 31 years with flexion allowing traditional greetings.

**Table 1:** Breakdown according to functional score.

PMA score	Number of patients	Percentage
16	1	7,1%
15	8	57,1%
14	5	35,7%
Total	14	100,0%

**Table 2:** Functional result according to indication.

PMA score	Complications of osteosynthesis / arthroplasty	Pseudarthrosis of the neck	AON	Total
16	0	0	1	1
15	2	6	1	9
14	1	0	3	4
Total	3	6	5	14
